# Induction chemoimmunotherapy versus radiotherapy alone for inoperable esophageal squamous cell carcinoma: a real-world study

**DOI:** 10.3389/fimmu.2026.1831726

**Published:** 2026-07-28

**Authors:** Junhui Wang, Jianxin Chen

**Affiliations:** Department of International Ward, Quzhou People′s Hospital, The Quzhou Affiliated Hospital, Wenzhou Medical University, Zhejiang, China

**Keywords:** esophageal squamous cell carcinoma, immunotherapy, induction therapy, overall survival, radiotherapy

## Abstract

**Background:**

For patients with locally advanced esophageal squamous cell carcinoma (ESCC) ineligible for surgery, definitive radiotherapy (RT) is the primary curative-intent treatment, yet outcomes remain suboptimal. The benefit of adding induction immunotherapy combined with chemotherapy prior to RT in this setting is unclear. This real-world study compared survival and safety between induction chemoimmunotherapy followed by RT and RT alone.

**Methods:**

This single-center, retrospective cohort study enrolled 159 patients with inoperable locally advanced ESCC treated between May 2021 and December 2025. Patients were categorized into an induction treatment group (n=48) receiving PD-1 inhibitor-based chemoimmunotherapy before RT, and a no-induction (control) group (n=111) receiving RT alone. Details regarding unsystematically administered concurrent chemotherapy were not captured. The primary endpoints were progression-free survival (PFS) and overall survival (OS). Toxicity was assessed per CTCAE v5.0.

**Results:**

Baseline characteristics were balanced. The induction group most commonly received camrelizumab (29.2%) with a taxane-based backbone. With a median follow-up, no significant survival difference was observed. Median PFS was 16.2 months (95% CI: 6.03-26.37) vs. 16.5 months (9.91-23.09) in the induction vs. control groups, respectively (P = 0.734). Median OS was 25.8 months (7.13-44.47) vs. 24.1 months (15.18-33.02), respectively (P = 0.659). Subgroup analyses showed no statistically significant treatment–subgroup interactions. Univariable analyses identified better nutritional status (NRS2002<3) as a favorable prognostic factor for both PFS and OS, and better performance status (ECOG PS 0-1) for OS. The induction group had significantly higher rates of Grade 3–4 adverse events, including neutropenia (60.4% vs. 7.2%), anemia (47.9% vs. 9.9%), thrombocytopenia (27.1% vs. 1.8%), and nausea (83.3% vs. 6.3%).

**Conclusion:**

In this real-world cohort of inoperable locally advanced ESCC patients, adding induction chemoimmunotherapy before definitive radiotherapy was not associated with improved PFS or OS but led to a significantly increased burden of severe toxicities compared to RT alone. These findings do not support the routine use of this intensive sequential strategy in an unselected, often frail, inoperable population.

## Introduction

Esophageal carcinoma (EC) ranks as the seventh most commonly diagnosed cancer and the sixth leading cause of cancer-related mortality worldwide, with squamous cell carcinoma (ESCC) being the predominant histological subtype, particularly in Eastern Asia (1). The management of locally advanced ESCC poses a significant clinical challenge. For patients with resectable disease, neoadjuvant chemoradiotherapy followed by surgery represents the standard of care, as established by the CROSS trial and reaffirmed by subsequent studies (2, 3). This multimodal approach has demonstrably improved survival outcomes compared to surgery alone.

However, a substantial proportion of patients with locally advanced ESCC are deemed ineligible for curative surgical resection in real-world clinical practice. This decision may stem from a multitude of factors, including advanced patient age, the presence of significant comorbidities (e.g., cardiopulmonary dysfunction), poor performance status, technically unresectable disease due to local invasion, or patient refusal (4). This “non-operative” cohort represents a common yet therapeutically complex population. For these patients, definitive concurrent chemoradiotherapy is the standard treatment for those who can tolerate it, whereas definitive radiotherapy alone may be used in selected patients who are unsuitable for concurrent chemotherapy. While effective, the long-term outcomes with definitive chemoradiotherapy remain suboptimal, with 5-year overall survival rates typically ranging from 20% to 35%, highlighting a pressing need for therapeutic advances (5, 6).

The advent of immunotherapy, particularly immune checkpoint inhibitors (ICIs) targeting the programmed death-1 (PD-1)/programmed death-ligand 1 (PD-L1) axis, has revolutionized the treatment landscape for advanced ESCC (7, 8). The success of ICIs in the metastatic setting has logically spurred investigation into their integration into earlier stages of disease. In the perioperative setting for resectable ESCC, the benefit of immunotherapy is increasingly recognized across different treatment pathways. Beyond the definitive chemoradiotherapy setting explored in trials such as KEYNOTE-975 (9), the CheckMate 577 trial specifically established adjuvant nivolumab as a new standard for patients with residual disease after neoadjuvant chemoradiotherapy and surgery (10). These data, spanning both perioperative and definitive CRT settings, strongly support the potent anti-tumor activity of ICIs in ESCC.

This success has led to the intuitive hypothesis that incorporating immunotherapy into the definitive, non-surgical management of locally advanced ESCC could yield similar benefits. The synergistic potential of immunotherapy with chemoradiotherapy is biologically plausible: chemotherapy and radiotherapy can induce immunogenic cell death, increase tumor antigen exposure, and modulate the tumor microenvironment, potentially enhancing the efficacy of subsequent or concurrent ICIs (11, 12). Consequently, the strategy of using induction (or neoadjuvant) immunotherapy combined with chemotherapy prior to definitive radiotherapy has garnered considerable interest. The goal is to achieve early systemic cytoreduction, eradicate micro-metastases, and potentially sensitize the primary tumor to subsequent radiotherapy, thereby improving ultimate disease control.

Nevertheless, the clinical value of this sequential approach, specifically, induction chemoimmunotherapy followed by definitive radiotherapy, in the inoperable locally advanced ESCC population remains ambiguous and is a subject of active debate. Existing evidence is fragmented and primarily derived from small-scale, single-arm phase II trials or retrospective studies with heterogeneous designs. Some studies suggest promising response rates and survival trends, while others report increased toxicity without a clear survival advantage. For instance, a phase II trial investigated iparomlimab and tuvonralimab combined definitive chemoradiotherapy in local advanced ESCC, showing encouraging activity (13). Furthermore, the landmark randomized controlled trials (RCTs) that established new standards, like KEYNOTE-975, predominantly enrolled patients *scheduled for surgery*, making their results not directly generalizable to the inherently different population of patients who are medically or technically inoperable (9). This latter group is often older, has a higher burden of comorbidities, and may have poorer performance status and nutritional status, factors that could significantly influence treatment tolerance, safety, and ultimate efficacy (14–16).

This gap between the promising data in surgical candidates and the uncertainty in non-surgical candidates underscores a critical need for high-quality, real-world evidence (RWE). Randomized controlled trials, the gold standard for efficacy, often have strict eligibility criteria that may exclude the very patients commonly encountered in clinics, such as the elderly or those with poor ECOG performance status (17). Real-world studies, therefore, play an indispensable complementary role. They assess the effectiveness and safety of therapeutic strategies as applied in routine clinical practice, capturing outcomes in broader, more representative patient populations, including those under-represented in RCTs (18, 19). The toxicity profile of induction chemoimmunotherapy, especially when sequenced with radical radiotherapy, is a major practical concern that can be thoroughly evaluated in a real-world setting.

Specifically, for the common clinical scenario of a patient with locally advanced ESCC who is not a surgical candidate, a fundamental and unresolved question persists: Does the addition of induction immunotherapy combined with chemotherapy prior to definitive radiotherapy confer a meaningful survival benefit over proceeding directly to radiotherapy, and if so, at what cost in terms of toxicity? The balance between potential efficacy gain and the risk of increased adverse events, which could compromise treatment delivery or quality of life, is unclear. Currently, there is a lack of large-scale, comparative real-world studies specifically designed to address this question in a balanced cohort.

To fill this evidence gap, we conducted this retrospective, real-world cohort study, aiming to compare the survival outcomes and safety profiles between two treatment strategies: 1) induction therapy with a combination of immunotherapy and chemotherapy followed by definitive radiotherapy, and 2) definitive radiotherapy alone, in patients with locally advanced ESCC who were deemed ineligible for surgical resection. We hypothesized that, in this real-world setting, the induction chemoimmunotherapy approach might be associated with different efficacy and toxicity compared to upfront radiotherapy. By analyzing a consecutive cohort of patients managed in daily practice, this study seeks to provide contemporary, pragmatic insights into the value of induction immunotherapy in this specific and prevalent patient population, thereby informing clinical decision-making and guiding the design of future prospective studies.

## Methods

### Study design and patient population

This was a single-center, retrospective, real-world cohort study conducted at Quzhou People′s Hospital. The study protocol was reviewed and approved by the ethical committee of the People’s Hospital of Quzhou, and the requirement for informed consent was waived due to the retrospective nature of the analysis, in accordance with national regulations and the Declaration of Helsinki.

We screened the electronic medical records of patients diagnosed with esophageal squamous cell carcinoma (ESCC) between May 1^st^ 2021 and December 30^th^ 2025. Patients were eligible for inclusion if they met the following criteria: (1) histologically confirmed diagnosis of locally advanced ESCC; (2) deemed ineligible for curative surgical resection by oncologists assessment due to comorbidities, advanced age, patient refusal, or technically unresectable disease; (3) received definitive radiotherapy (RT) as a primary local treatment modality, with or without prior induction therapy; (4) had complete medical records regarding treatment details, follow-up, and outcomes. Key exclusion criteria were: (1) presence of distant metastases (M1 disease) at initial diagnosis, except for patients with supraclavicular lymph node metastasis who were still considered for definitive RT; (2) previous history of other malignancies within the past 5 years; (3) receipt of any prior systemic therapy or radiotherapy for ESCC before the initiation of the treatment sequences defined in this study; (4) incomplete treatment records or loss to follow-up immediately after diagnosis.

### Treatment regimens

Based on the initial therapeutic strategy documented in the medical records, enrolled patients were categorized into two groups for comparative analysis; treatment allocation was not randomized but was determined by the treating oncologists in routine clinical practice according to patients’ general condition, comorbidities, disease status, expected treatment tolerance, and patient preference.

#### Induction treatment group

Patients in this group received induction therapy comprising a combination of immunotherapy and chemotherapy prior to the initiation of definitive radiotherapy. The choice of specific immune checkpoint inhibitor (ICI) and chemotherapy regimen was at the discretion of the treating oncologist, reflecting real-world clinical practice. Immunotherapy agents used included programmed cell death protein 1 (PD-1) inhibitors such as camrelizumab, toripalimab, sintilimab, tislelizumab, serplulimab, and pembrolizumab. The concomitant chemotherapy backbone was primarily taxane-based (e.g., paclitaxel or docetaxel), with one patient receiving a fluoropyrimidine-based regimen. The number of cycles of induction therapy administered was recorded.

#### No-induction treatment group (control group)

Patients in this group proceeded directly to definitive radiotherapy, without receiving any systemic induction therapy beforehand.

Notably, the decision to administer concurrent chemotherapy alongside radiotherapy was individualized based on patient tolerance and was not systematically recorded as a pre-specified variable in this study. Notably, the decision to administer concurrent chemotherapy alongside radiotherapy was individualized based on patient tolerance; however, concurrent chemotherapy was not systematically recorded as a pre-specified variable in this study. As such, we cannot assess the potential impact of concurrent chemotherapy on survival outcomes, nor can we verify its balance between the two treatment groups. The specific radiotherapy techniques (e.g., intensity-modulated radiotherapy), total dose, fractionation, and target volume delineation were implemented in strict accordance with institutional standard protocols for esophageal cancer radiotherapy. Individual treatment plans were customized based on each patient’s tumor characteristics, disease extent, and treatment tolerance, and raw plan details were retrieved from electronic medical records. Given the retrospective design, specific parameters such as dose to organs at risk were not uniformly available; however, key prescription parameters are provided in **Supplementary Table 1** to demonstrate the broad comparability of radiotherapy delivery between the two groups. Notably, while the full cohort included 159 patients, complete radiotherapy records were unavailable for 3 patients in the induction treatment group and 8 patients in the no-induction treatment group; thus, data in **Supplementary Table 1** reflect the 148 patients with available information.

### Data collection and study variables

Demographic and clinical data were extracted from the hospital information system. Collected baseline variables included: age, gender, smoking history, drinking history, Eastern Cooperative Oncology Group Performance Status (ECOG PS), and Tumor-Node-Metastasis (TNM) stage according to the American Joint Committee on Cancer (AJCC) 8th edition. Nutritional risk was assessed using the Nutritional Risk Screening 2002 (NRS2002) tool, with a score of ≥3 indicating nutritional risk. Pre-treatment serum lactate dehydrogenase (LDH) level was also recorded as a potential biomarker.

Treatment-related variables for the induction group included the specific ICI agent, number of immunotherapy cycles, and the chemotherapy regimen. For all patients, details regarding radiotherapy (dose, technique) were noted.

### Study endpoints and assessments

The primary endpoints of this study were progression-free survival (PFS) and overall survival (OS). PFS was defined as the time from the initiation of the first treatment (start of induction therapy for the induction group or start of radiotherapy for the no-induction group) to the first documented disease progression (based on radiological imaging per RECIST 1.1 criteria as assessed by the treating physicians or a dedicated radiologist at our institution during routine follow-up) or death from any cause, whichever occurred first. OS was defined as the time from treatment initiation to death from any cause. Patients without an event at the last follow-up were censored. Secondary endpoints included the safety and toxicity profiles of the treatment strategies. Adverse events (AEs) were graded according to the Common Terminology Criteria for Adverse Events (CTCAE) version 5.0. All documented AEs occurring from the start of any treatment component until 90 days after the last treatment were collected and analyzed.

### Statistical analysis

Continuous variables are presented as mean ± standard deviation (SD) or median with interquartile range as appropriate. Categorical variables are described as frequencies and percentages. To assess the comparability of the two treatment groups at baseline, differences in demographic and clinical characteristics were evaluated using the Chi-square test or Fisher’s exact test for categorical variables, and the Student’s t-test or Mann-Whitney U test for continuous variables, as applicable. A two-sided p-value of <0.05 was considered statistically significant. Survival curves for PFS and OS were generated using the Kaplan-Meier method, and differences between groups were compared with the log-rank test. The median survival times with their corresponding 95% confidence intervals (CIs) were reported. To explore potential differential treatment effects across clinically relevant subpopulations, pre-specified subgroup analyses for both PFS and OS were performed. Subgroups were defined by age (<65 vs. ≥65 years), gender, TNM stage (I-II vs. III-IV), smoking history (yes vs. no), ECOG PS (0–1 vs. 2), and serum LDH level (using a cutoff of 250 U/L, the cutoff value for serum LDH was selected based on the upper limit of normal at our institution). Hazard ratios (HRs) with 95% CIs for the treatment effect (induction vs. no-induction) within each subgroup were calculated using Cox proportional hazards models, and interaction tests were conducted to evaluate heterogeneity of treatment effect across subgroups. Furthermore, univariable Cox proportional hazards regression analyses were employed to evaluate the associations between baseline clinical characteristics (including age, gender, TNM stage, smoking history, drinking history, ECOG PS, NRS2002 score, and LDH level) and survival outcomes (PFS and OS) in the entire cohort. Results are presented as HRs with 95% CIs. Given the sample size and the number of events, a multivariable Cox regression analysis was not performed to identify independent prognostic factors, as the study was primarily designed for treatment comparison. In addition, due to the retrospective design, detailed data regarding the administration of concurrent chemotherapy in the no-induction group were incompletely recorded. Without this critical variable, attempts at propensity-score matching or multivariable adjustment could introduce bias rather than mitigate it; thus, these analyses were not performed. The univariable analyses are presented for exploratory purposes. All statistical analyses were performed using SPSS Statistics, version 23.0; IBM Corp., Armonk, NY, USA.

## Results

### Patient enrollment and baseline characteristics

**Figure 1:**
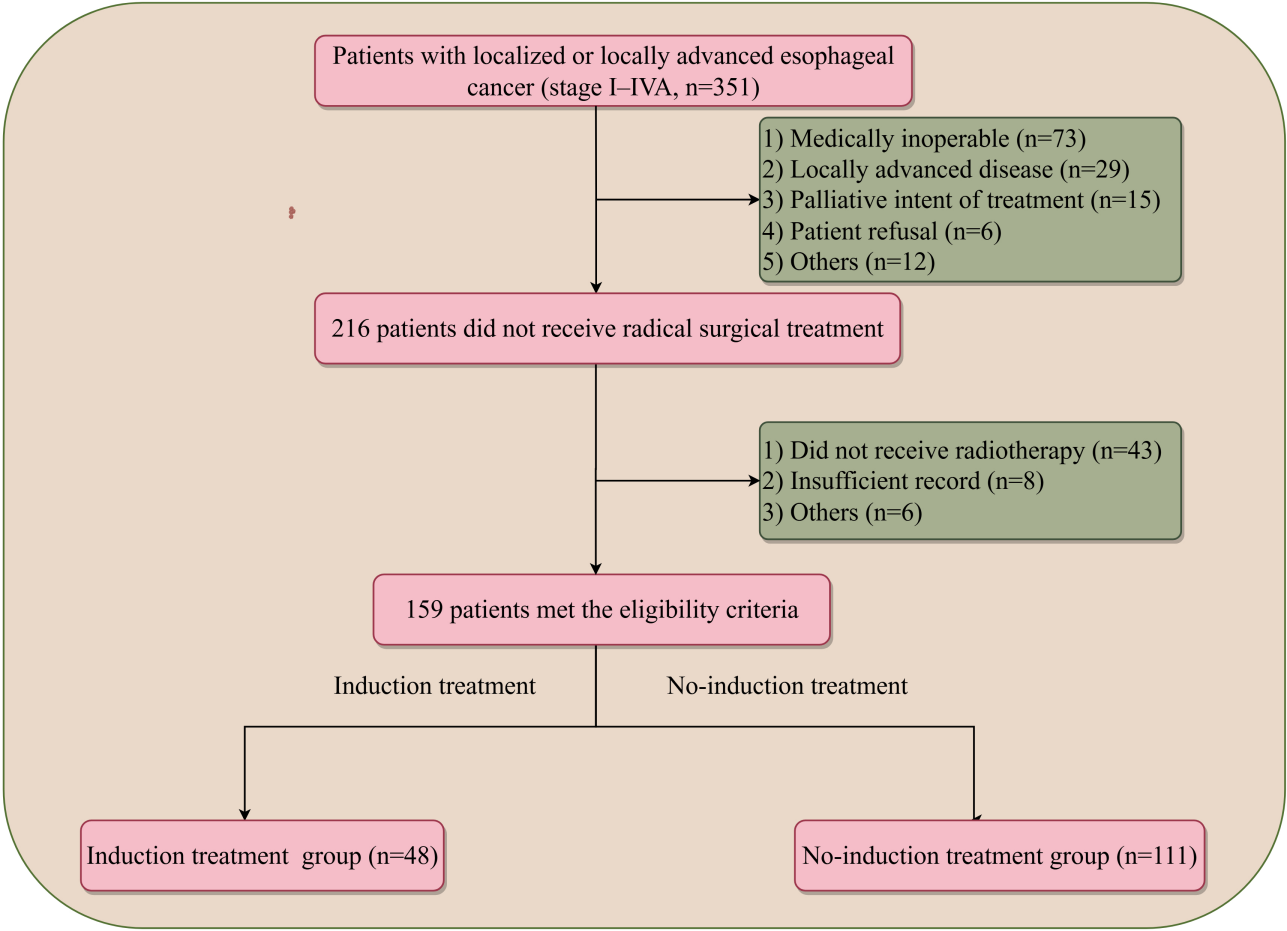
Patient selection flowchart.

A total of 159 patients with locally advanced esophageal squamous cell carcinoma (ESCC) deemed ineligible for surgery were retrospectively enrolled in this real-world study. The patient screening and inclusion process is detailed in a consort flow diagram (**Figure 1**). Based on the initial therapeutic strategy, patients were categorized into two groups: an induction treatment group (n=48) who received immunotherapy combined with chemotherapy prior to definitive radiotherapy, and a no-induction treatment group (n=111) who proceeded directly to radiotherapy.

The baseline demographic and clinical characteristics of the two groups are summarized in **Table 1**. The median age of the cohort was skewed towards older patients, with 136 patients (85.5%) aged 65 years or older. The majority were male (129 patients, 81.1%). Most patients presented with advanced disease, as 85 patients (53.5%) had TNM stage III or IV disease. A substantial proportion had a smoking history (79 patients, 49.7%) and a drinking history (69 patients, 43.4%). Nutritional risk, assessed by NRS2002 (score ≥3), was high and comparable between groups (127 patients, 79.9%). The performance status was generally poor, with 121 patients (76.1%) having an ECOG PS of 2. Serum lactate dehydrogenase (LDH) levels were similar between the cohorts. Statistical analysis confirmed no significant differences between the induction and no-induction groups across all recorded baseline characteristics, including age (P = 0.604), gender (P = 0.364), TNM stage (P = 0.615), smoking history (P = 0.523), drinking history (P = 0.269), NRS2002 score (P = 0.884), ECOG PS (P = 0.317), and LDH levels (P = 0.596), indicating the groups were well-balanced for comparative analyses.

**Table 1:** Baseline characteristics (n=159).

Baseline characteristics	Induction treatment group (n=48)	No-induction treatment group (n=111)	*P*
Age, years			
Median (range)	73 (54-83)	77 (51-92)	
Age group, n (%)			0.604
≥65	40 (83.3)	96 (86.5)	
< 65	8 (16.7)	15(13.5)	
Gender, n (%)			0.364
Male	41 (85.4)	88 (79.3)	
Female	7 (14.6)	41 (20.7)	
TNM stage, n (%)			
I	5 (10.4)	11 (9.9)	0.615
II	17 (35.4)	41 (36.9)	
III	20 (41.7)	52 (46.8)	
IV	6 (12.5)	7 (6.4)	
Smoking history, n (%)			0.523
Yes	22(45.8)	57 (51.4)	
No	26 (54.2)	54 (48.6)	
Drinking history, n (%)			0.269
Yes	24 (50)	45 (40.5)	
No	24 (50)	66 (59.5)	
NRS2002, n (%)			0.884
≥3	38 (79.2)	89 (80.2)	
<3	10 (20.8)	22 (19.8)	
ECOG PS, n (%)			0.317
0–1	9(18.8)	29 (26.1)	
2	39 (81.2)	82 (73.9)	
LDH (U/L), Mean ± SD	199.68 ± 46.24	205.00 ± 62.41	0.596

LDH, Lactate Dehydrogenase; ECOG PS, Eastern Cooperative Oncology Group Performance Status; SD, Standard Deviation; NRS 2002, Nutritional Risk Screening 2002.

### Baseline demographic and clinical characteristics

### Details of the induction treatment regimens

The specific regimens for the 48 patients receiving induction therapy are detailed in **Table 2**. Regarding immunotherapy agents, camrelizumab was the most frequently used (14 patients, 29.2%), followed by toripalimab (9 patients, 18.8%), sintilimab and tislelizumab (8 patients each, 16.7%), serplulimab (5 patients, 10.4%), and pembrolizumab (4 patients, 8.3%). Patients received a mean of 2.91 (± 2.25 SD) cycles of immunotherapy prior to radiotherapy. The concomitant chemotherapy backbone was overwhelmingly taxane-based, administered to 47 patients (97.9%), with only one patient (2.1%) receiving a fluoropyrimidine-based regimen.

**Table 2:** Details of induction treatment group regimens (n=48).

Regimens	n (%)
Immunotherapy agent	
Camrelizumab	14 (29.2)
Toripalimab	9 (18.8)
Sintilimab	8 (16.7)
Tislelizumab	8 (16.7)
Serplulimab	5 (10.4)
Pembrolizumab	4 (8.3)
Cycles of immunotherapy, mean ± SD	2.91 ± 2.25
Chemotherapy regimen, n (%)	
Taxane-based chemotherapy	47 (97.9)
Fluoropyrimidine-based chemotherapy	1 (2.1)

### Survival outcomes

**Figure 2:**
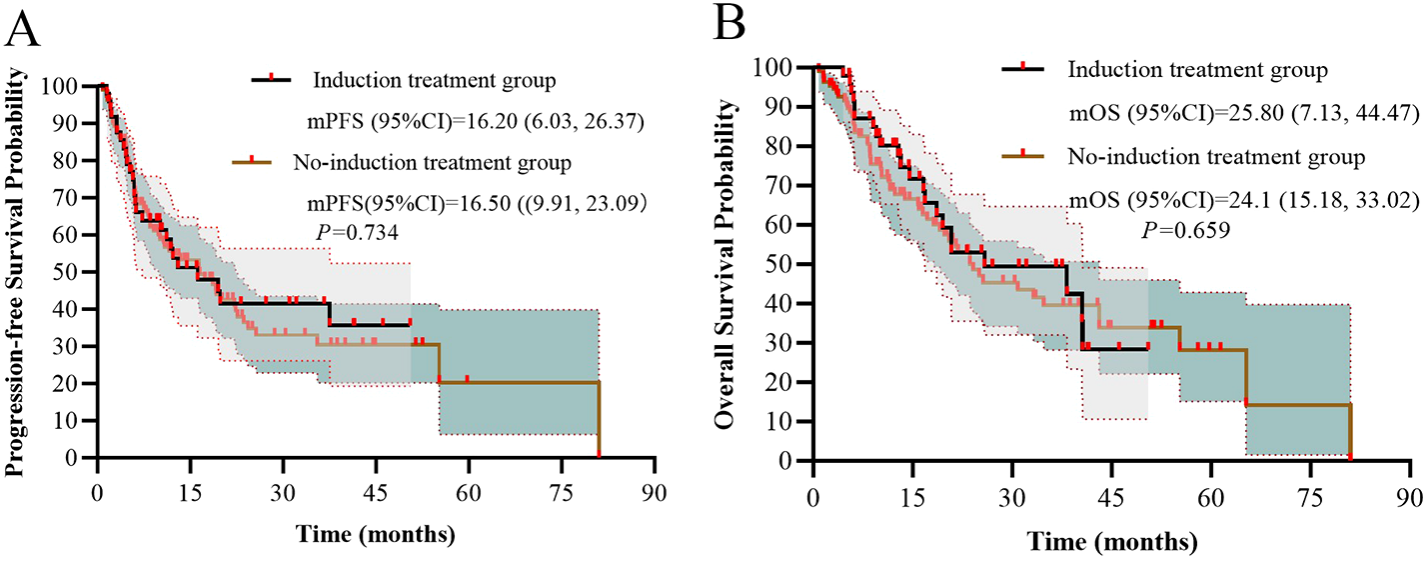
Kaplan-Meier Curves of the comparisons for PFS **(A)** and OS **(B)** between induction treatment group and no-induction group.

With a median follow-up of 28.2 months (range: 2.3–46.0 months) in the induction group and 28.6 months (range: 2.0–46.4 months) in the no-induction group (P = 0.791 by Mann-Whitney U test), the dataset demonstrated adequate data maturity to support valid survival comparisons. Survival outcomes between the two treatment groups were evaluated. Kaplan-Meier curves for progression-free survival (PFS) and overall survival (OS) are presented in **Figure 2**. The log-rank test revealed that there was no statistically significant difference in PFS between the induction immunotherapy-chemotherapy group and the radiotherapy-alone group (P = 0.734). The median PFS was 16.2 months (95% CI: 6.03-26.37) in the induction group versus 16.5 months (95% CI: 9.91-23.09) in the no-induction group. Similarly, the comparison of OS also showed no statistically significant difference (P = 0.659). The median OS was 25.8 months (95% CI: 7.13-44.47) in the induction group compared to 24.1 months (95% CI: 15.18-33.02) in the control group. These results indicate that, in this real-world cohort, the strategy of adding induction immunotherapy combined with chemotherapy prior to radiotherapy did not demonstrate a significant improvement in survival outcomes compared to proceeding directly to radiotherapy.

### Subgroup analyses of survival

**Figure 3:**
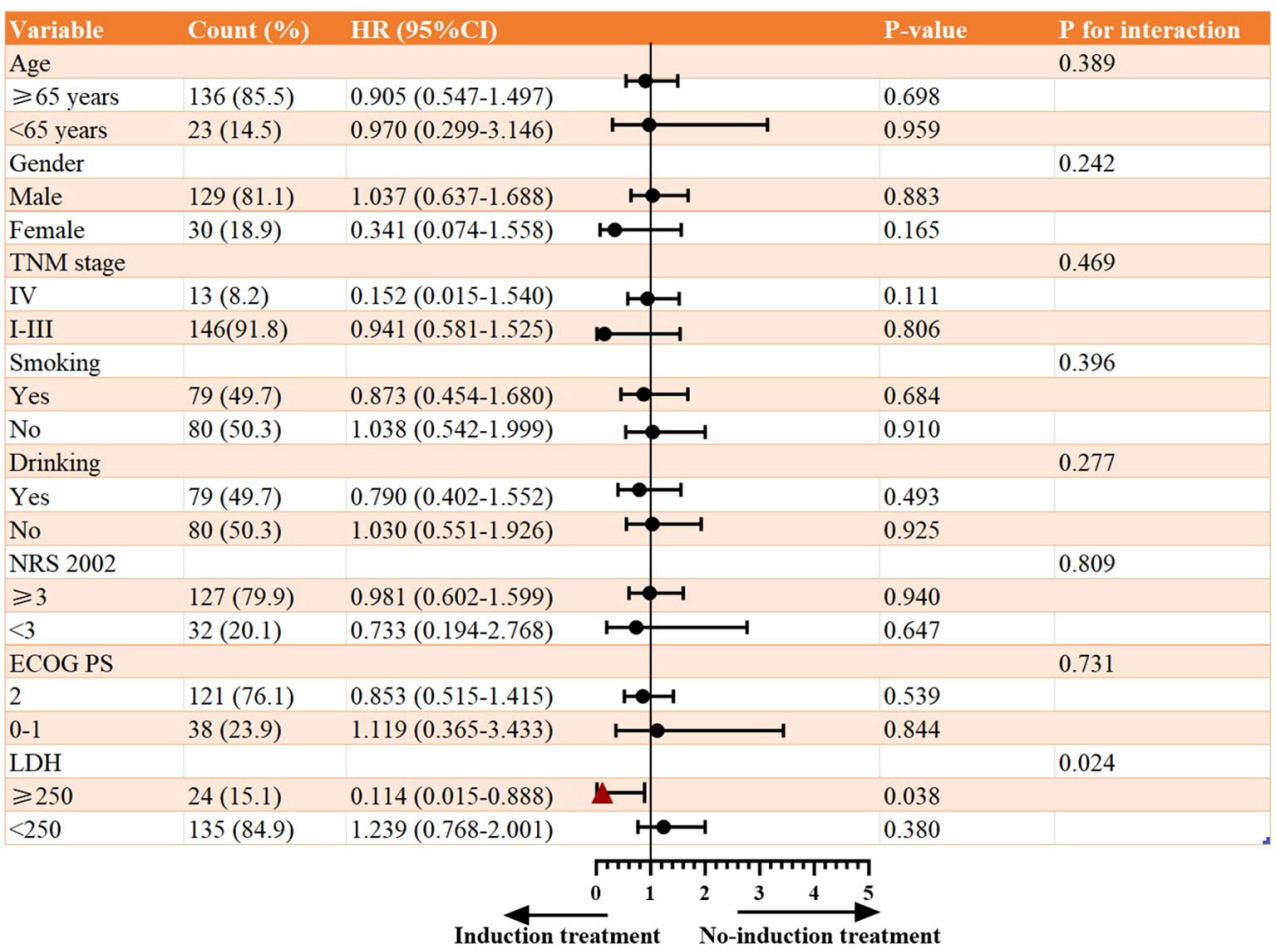
Subgroup analysis of PFS.

**Figure 4:**
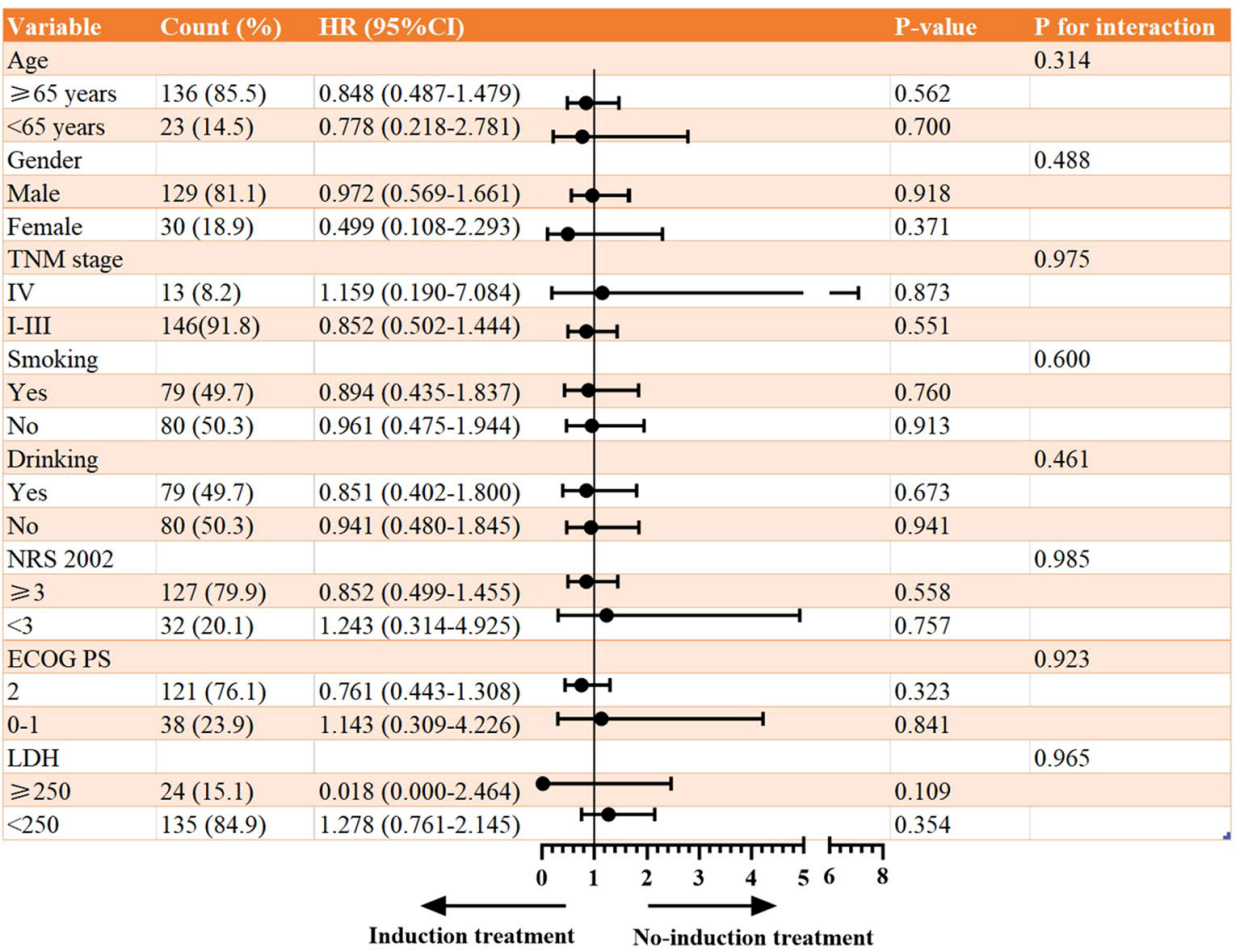
Subgroup analysis of OS.

Subgroup analyses for progression-free survival (PFS) and overall survival (OS) were performed based on key clinical factors, with the results presented in forest plots (**Figure 3** for PFS, **Figure 4** for OS). The analyses included subgroups defined by age (<65 vs. ≥65 years), gender, TNM stage (I-II vs. III-IV), smoking history, ECOG performance status (0–1 vs. 2), and serum lactate dehydrogenase (LDH) level.

The forest plots demonstrated that, for the vast majority of the examined subgroups, the treatment effect (induction therapy vs. no induction) did not reach statistical significance. The hazard ratios (HR) for both PFS and OS across subgroups defined by age, gender, TNM stage, smoking history, and ECOG PS were not statistically significant, as their 95% confidence intervals (CI) included 1.0. This indicates a lack of statistically significant differential treatment benefit within these specific patient subsets in this study.

In exploratory subgroup analyses, an isolated PFS signal was observed in the small subgroup of patients with elevated serum LDH (>250 U/L) who received induction therapy (HR 0.114, 95% CI: 0.015-0.888, P = 0.038). No such benefit was seen for OS in this subgroup (HR 0.018, 95% CI: 0.000-2.464, P = 0.109). The formal interaction test for the LDH subgroup was not statistically significant (P-interaction >0.05), and no corresponding OS benefit was observed. Therefore, this isolated PFS signal should be interpreted as exploratory and hypothesis-generating only, particularly given the very small sample size and the issue of multiple subgroup comparisons. Formal interaction tests for the listed subgroups were non-significant (all P for interaction > 0.05), suggesting no strong evidence of heterogeneity in treatment effect across these different patient characteristics, aside from the noted observation in the LDH subgroup for PFS.

### Univariate cox regression analyses

**Figure 5:**
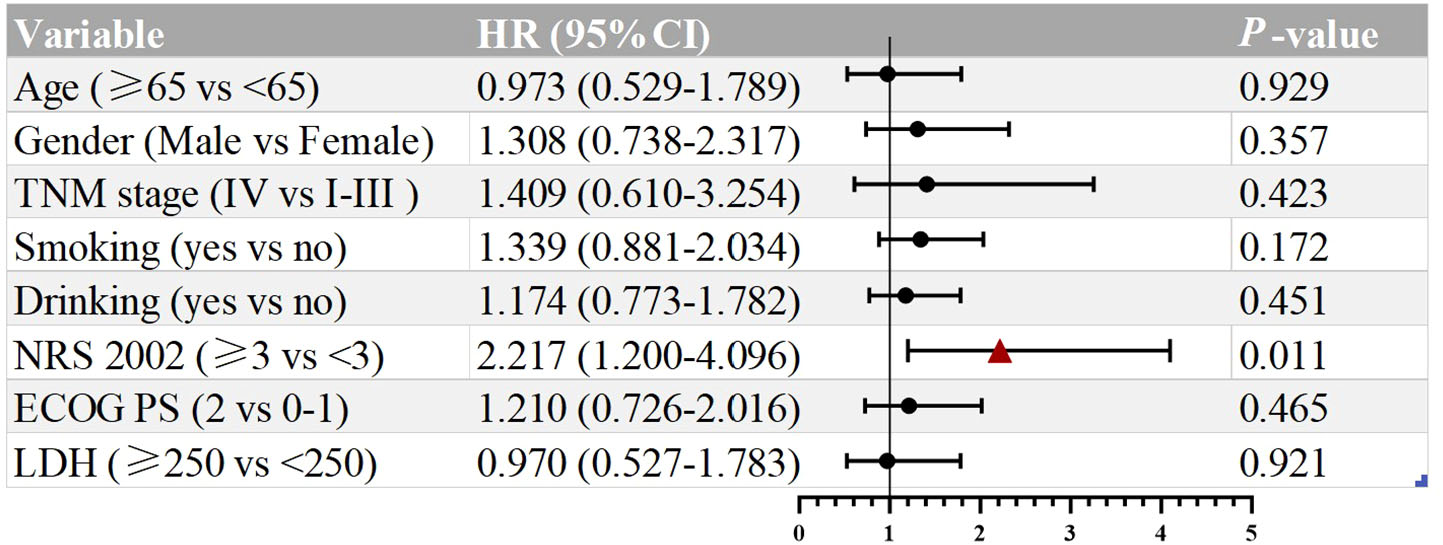
Univariate Cox Regression Analysis for PFS.

**Figure 6:**
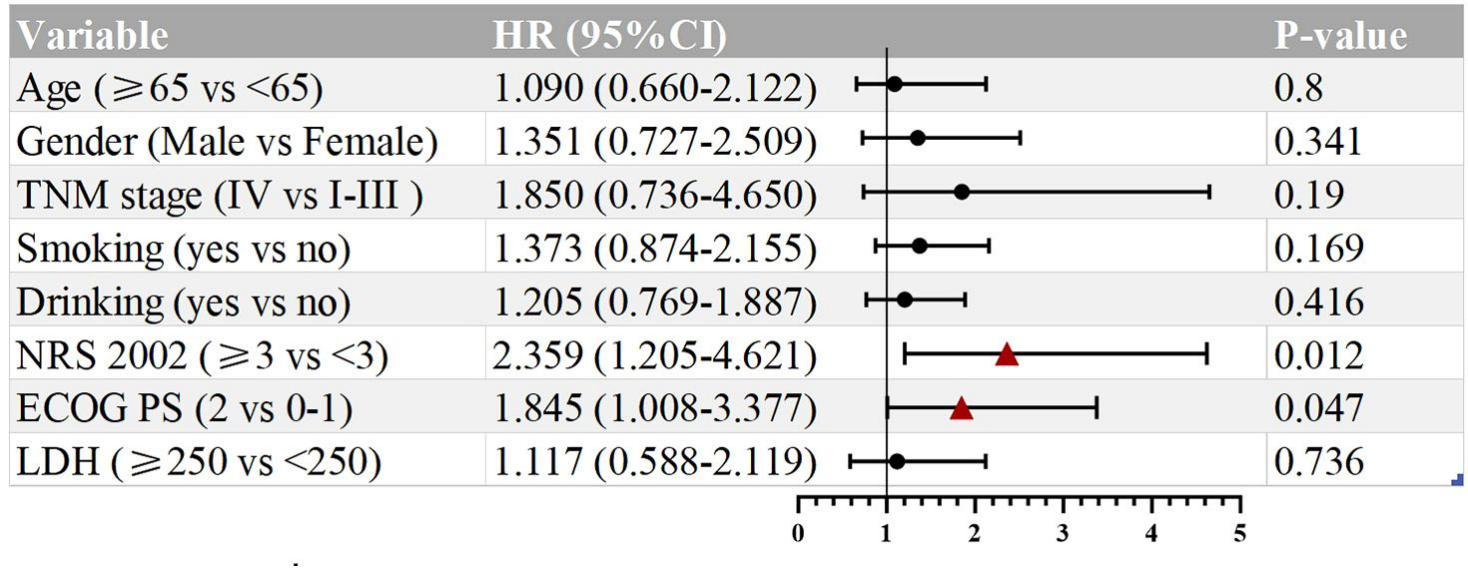
Univariate Cox Regression Analysis for OS.

Univariable Cox proportional hazards regression analyses were conducted to evaluate the association between baseline clinical characteristics and survival outcomes in the entire cohort. The results for progression-free survival (PFS) and overall survival (OS) are summarized in forest plots (**Figures 5**, **6**, respectively). For PFS, the analysis identified nutritional status, as assessed by the NRS2002 score, as a significant prognostic factor. Patients with a lower nutritional risk (NRS2002 score <3) demonstrated a significantly longer PFS compared to those with a higher nutritional risk (NRS2002 score ≥3), with a HR of 2.217 (95% CI: 1.200-4.096, P = 0.011). None of the other included variables, including age, gender, TNM stage, smoking history, drinking history, ECOG performance status, and serum lactate dehydrogenase (LDH) level, showed a statistically significant association with PFS in this univariable analysis. For OS, two factors emerged as significant prognostic indicators. First, a better nutritional status (NRS2002 score <3) was significantly associated with improved OS (HR: 2.359, 95% CI: 1.205-4.621; P = 0.012). Second, a better performance status (ECOG PS of 0-1) was also significantly associated with longer OS compared to a poorer performance status (ECOG PS of 2) (HR 1.845, 95% CI: 1.008-3.377; P = 0.047). In contrast, variables such as age, gender, TNM stage, smoking history, drinking history, and serum LDH level did not show a statistically significant association with OS in the univariable model.

In summary, the univariable analyses highlighted that better baseline nutritional status (NRS2002 score <3) was a favorable prognostic factor for both PFS and OS in this patient population. Additionally, a better performance status (ECOG PS 0-1) was specifically associated with improved OS. Other common clinical parameters, including disease stage, did not reach statistical significance as prognostic factors in this analysis.

### Adverse events

The safety profiles, with adverse events graded per CTCAE criteria, are detailed in **Table 3**. The induction treatment group experienced a higher incidence of treatment-related adverse events. Hematological toxicities were notably more frequent and severe. Grade 3–4 anemia occurred in 23 patients (47.9%) in the induction group versus 11 patients (9.9%) in the control group. Grade 3–4 neutropenia was observed in 29 induction patients (60.4%) compared to 8 control patients (7.2%). Grade 3–4 thrombocytopenia was also more common with induction therapy (13 patients, 27.1% vs. 2 patients, 1.8%).

**Table 3:** Adverse events (n=159).

Adverse events	Induction treatment group (n=48)		No-induction treatment group (n=111)		P-value for Grade 3–4
	Grade 1–2, n (%)	Grade 3–4, n (%)	Grade 1–2, n (%)	Grade 3–4, n (%)	
Anemia	43 (89.6)	23 (47.9)	59 (53.2)	11 (9.9)	<0.001
Thrombocytopenia	19 (39.9)	13 (27.1)	12 (10.8)	2 (1.8)	<0.001
Fatigue	39 (81.3)	26 (54.2)	63 (56.8)	19 (17.1)	<0.001
Nausea	15 (31.3)	40 (83.3)	17 (15.3)	7 (6.3)	<0.001
Rash	11 (22.9)	2 (4.2)	6 (5.4)	1 (0.9)	0.217
Neutropenia	41 (85.4)	29 (60.4)	27 (24.3)	8 (7.2)	<0.001
Radiation pneumonitis	14 29.2)	0	29 (26.1)	3 (2.7)	0.554
Weight loss	17 (35.4)	6 (12.5)	76 (68.5)	19 (17.1)	0.636
Diarrhea	8 (16.7)	3 (6.3)	9 (8.1)	2 (1.8)	0.162
Mucositis/stomatitis	13 (27.1)	3 (6.3)	22 (19.8)	7 (6.3)	1.000
Neuropathy	37 (77.1)	16 (33.3)	4 (3.6)	0	<0.001
Cardiotoxicity	5 (10.4)	0	2 (1.8)	0	——

Among non-hematological toxicities, severe (grade 3-4) nausea was markedly high in the induction group (40 patients, 83.3%) versus the control group (7 patients, 6.3%). Fatigue (grade 3-4: 26 [51.2%] vs. 19 [17.1%]), neuropathy (any grade: 37 [77.1%] vs. 4 [3.6%]), and rash were more prevalent with induction therapy. Interestingly, weight loss of any grade was more frequently reported in the no-induction group (95 patients, 85.6%) compared to the induction group (23 patients, 47.9%). The rates of radiation pneumonitis, diarrhea, mucositis/stomatitis, and cardiotoxicity were generally low and comparable between groups. No grade 3–4 radiation pneumonitis or cardiotoxicity was reported in the induction treatment group.

## Discussion

This real-world cohort study provides a comparative assessment of induction chemoimmunotherapy followed by definitive radiotherapy versus radiotherapy alone in a cohort of 159 patients with locally advanced ESCC deemed ineligible for surgical resection. The primary finding is that, within the context of routine clinical practice, the addition of induction therapy with PD-1 inhibitors combined with chemotherapy prior to radiotherapy did not confer a statistically significant improvement in either progression-free survival (median PFS: 16.2 vs. 16.5 months, P = 0.734) or overall survival (median OS: 25.8 vs. 24.1 months, P = 0.659) compared to proceeding directly to radiotherapy. This null result in survival benefit was accompanied by a markedly increased burden of treatment-related adverse events, particularly hematological toxicities and severe nausea, in the induction therapy group. These findings offer critical, practice-informing insights into the risk-benefit profile of this sequential treatment strategy in a prevalent yet understudied patient population.

The induction approach was selected in routine practice because it could potentially reduce tumor burden before radiotherapy, provide early systemic disease control, and allow assessment of treatment tolerance before definitive local therapy. The absence of a survival advantage with induction chemoimmunotherapy in our study stands in contrast to the paradigm established in the perioperative setting for resectable ESCC, where the integration of immunotherapy has led to practice-changing improvements in pathological responses and survival (10, 20). This discrepancy highlights the fundamental differences between surgical and non-surgical management paradigms and cautions against the direct extrapolation of benefits from one setting to the other. In the definitive non-operative setting, radiotherapy serves as the cornerstone local modality, and its efficacy might be constrained by intrinsic radioresistance or the advanced disease biology in this often frail population. Our findings, however, are consistent with the outcomes reported in several recent investigations focusing on immunotherapy combined with definitive chemoradiotherapy for inoperable locally advanced ESCC. For instance, a multicenter real-world study published in 2025 comparing definitive chemoradiotherapy combined with anti-PD-1 immunotherapy to chemoradiotherapy alone found a significantly longer overall survival for the combination group, but also noted that after adjustment, there was no difference in overall survival among induction, concurrent, and consolidation immunotherapy subgroups (20). In contrast, our study directly comparing induction chemoimmunotherapy followed by RT versus RT alone in a similar population found no significant survival benefit (median PFS: 16.2 vs. 16.5 months, P = 0.734; median OS: 25.8 vs. 24.1 months, P = 0.659). These studies indicate that while immunotherapy combined with radiotherapy/chemoradiotherapy shows activity in inoperable ESCC, the magnitude of survival improvement may be limited, and the optimal timing of integration (induction, concurrent, or consolidation) remains undefined. In this context, the ongoing ARION UCGI 33/PRODIGE 67 trial by Modesto et al., which evaluates definitive CRT plus durvalumab followed by maintenance/consolidation durvalumab, may provide further evidence for concurrent and consolidation immunotherapy strategies in unresectable locally advanced esophageal cancer (21).

Several plausible explanations may account for the lack of survival difference. First, the patient population in this real-world study represents a challenging cohort, with a median age skewed towards the elderly (85.5% ≥65 years), a high prevalence of nutritional risk (79.9%), and a majority with poor performance status (76.1% ECOG PS 2). These factors are strongly associated with reduced treatment tolerance, compromised immune function, and overall poorer prognosis (14). The increased toxicity from induction therapy, as evidenced in our safety analysis, may have led to treatment delays, dose reductions, or compromised delivery of subsequent definitive radiotherapy, potentially offsetting any potential anti-tumor benefit. Second, the biological rationale for synergy, where chemotherapy/radiotherapy induces immunogenic cell death to prime a response enhanced by ICIs, might be insufficient to overcome the aggressive tumor biology or the immunosuppressive microenvironment in this specific setting. Furthermore, the duration and intensity of induction therapy in real-world practice are variable. In our cohort, patients received a mean of 2.91 cycles of immunotherapy, which may be suboptimal for achieving a deep and durable systemic immune activation compared to the fixed, often longer durations used in clinical trials.

The comprehensive subgroup analyses provided nuanced insights. Reassuringly, the treatment effect (induction vs. no induction) was largely consistent across most predefined subgroups, including age, gender, TNM stage, and ECOG PS, with no significant heterogeneity detected. This suggests that the overall null finding is broadly applicable across these common clinical dichotomies within our inoperable cohort. The most intriguing, though hypothesis-generating, finding was the statistically significant PFS benefit observed in the small subgroup of patients with elevated serum LDH (>250 U/L) who received induction therapy (HR 0.114, P = 0.038). Serum LDH is a well-known marker of aggressive tumor biology, high tumor burden, and anaerobic glycolysis, and is a negative prognostic factor in various cancers (22, 23). It is plausible that tumors with high metabolic activity and a potentially more inflamed microenvironment might be more susceptible to the combined effects of chemotherapy and immunotherapy (24). This aligns with biomarker analyses from advanced ESCC trials, where high TMB or inflammatory gene signatures sometimes correlate with ICI benefit (25). However, this observation must be interpreted with extreme caution due to the very small sample size in this biochemical subgroup, the lack of a corresponding significant OS benefit, and the inherent risk of false-positive findings from multiple subgroup comparisons. It underscores the need for prospective validation and further exploration of biomarkers, such as LDH, PD-L1 expression, or tumor mutational burden, to identify the minority of patients who might derive exceptional benefit from an intensified induction approach.

The univariable Cox regression analysis identified better nutritional status (NRS2002 score <3) as a significant favorable prognostic factor for both PFS and OS, and better performance status (ECOG PS 0-1) for OS. This reinforces the critical importance of comprehensive geriatric and nutritional assessment in managing older, inoperable ESCC patients. Malnutrition and poor performance status are powerful determinants of outcomes, likely influencing tolerance to therapy, risk of complications, and overall survival independent of the anticancer treatment received. Interestingly, TNM stage was not a significant prognostic factor in our analysis, possibly due to the uniformly advanced nature of the “inoperable” designation or the overwhelming prognostic weight of factors like nutrition and performance status in this specific population. These findings highlight that for clinicians, optimizing a patient’s general condition before and during treatment may be as crucial as the choice of anticancer regimen itself.

The safety analysis revealed a substantially higher toxicity burden associated with the induction chemoimmunotherapy strategy. The induction group experienced significantly higher rates of severe (Grade 3-4) hematological toxicities (neutropenia 60.4%, anemia 47.9%, thrombocytopenia 27.1%) and non-hematological toxicities like nausea (83.3%) and fatigue. The high rate of severe nausea is particularly notable and may be attributable to the combination of taxane-based chemotherapy with immunotherapy, though the exact mechanism warrants further study. These toxicities are consistent with the known profiles of the individual agents but underscore the additive effects when combined and sequenced before radical radiotherapy. The increased toxicity did not translate into a higher rate of classic radiation-induced toxicities like severe pneumonitis or cardiotoxicity, suggesting the safety concerns are primarily related to the systemic induction component. The higher incidence of any-grade weight loss in the no-induction group is an unexpected finding that may relate to disease progression or differences in supportive care, but it does not offset the clear and substantial increase in high-grade toxicities with induction therapy. This toxicity-efficacy calculus is central to clinical decision-making. In the absence of a survival benefit, the significantly increased risk of severe adverse events makes a routine recommendation for induction chemoimmunotherapy in this broad, unselected inoperable population difficult to justify.

Our study has several limitations inherent to its real-world, retrospective design. First, despite the lack of significant differences in recorded baseline characteristics, unmeasured confounding factors (e.g., detailed comorbidity indices, specific reasons for inoperability, socioeconomic factors) could have influenced both treatment selection and outcomes. The non-randomized assignment to treatment groups and the imbalance between the induction group and the no-induction group are key limitations, although they reflect real-world clinical decision-making. Second, because this was a retrospective real-world cohort with a fixed sample size and unequal group sizes, no formal *a priori* power calculation was performed, and the study may have been underpowered to detect modest but clinically meaningful differences in PFS or OS, especially in subgroup analyses. Third, the choice of immunotherapy agent and chemotherapy regimen was heterogeneous, reflecting real-world practice but introducing variability. Fourth, data on detailed radiotherapy parameters (e.g., dose to organs at risk), tumor location, and the reasons for omitting concurrent chemotherapy with radiotherapy were not fully captured, which could impact outcomes. In addition, a major limitation of this study is the lack of detailed information regarding concurrent chemotherapy in the control group. While baseline characteristics were balanced, the ‘no-induction’ group comprised 111 patients, among whom an undocumented proportion may have received concurrent systemic therapy. This heterogeneity within the control arm, combined with the smaller sample size of the induction group (n=48), may have impacted the robustness of our survival comparisons and represents a source of potential confounding that we were unable to adjust for statistically. Fifth, another limitation of this study is the lack of systematic data on concurrent chemotherapy administration. While baseline characteristics were balanced, the undocumented proportion of patients receiving concurrent systemic therapy in the no-induction group represents a source of potential confounding that we were unable to adjust for statistically. Finally, formal geriatric or frailty assessment data, such as G8 scores, and patient-reported quality of life (QoL) data were not available, which limited our ability to evaluate the impact of frailty and treatment-related burden in this elderly population. A deterioration in QoL due to increased toxicity without a survival gain would further argue against the routine use of this strategy.

In conclusion, this real-world study found that for patients with inoperable locally advanced ESCC, the strategy of adding induction chemoimmunotherapy prior to definitive radiotherapy, compared to radiotherapy alone, was associated with significantly increased treatment-related toxicity without a demonstrable improvement in progression-free or overall survival. The results suggest that the routine application of this intensive sequential approach in an unselected, often frail, inoperable population is not supported by current evidence. Clinical decision-making should therefore be highly individualized, weighing any potential benefit against the substantial risk of severe adverse events. Future prospective, ideally randomized trials are urgently needed to definitively answer this question. Such trials should incorporate rigorous biomarker exploration and comprehensive geriatric and QoL assessments to identify patients who are most likely to benefit from this demanding yet potentially promising therapeutic sequence.

## Data Availability

The raw data supporting the conclusions of this article will be made available by the authors, without undue reservation.
