# Supplementary material for: Induction chemoimmunotherapy versus radiotherapy alone for inoperable esophageal squamous cell carcinoma: a real-world study
Source: Front Immunol. 2026 Jul 28;17:1831726. doi: 10.3389/fimmu.2026.1831726 (PMC13457468; doi:10.3389/fimmu.2026.1831726)
Supplement: Supplementary file 1 [file DataSheet1.docx]

Supplementary Table S1. Descriptive statistics of radiotherapy parameters by treatment group.

| **Parameter** | **Induction Group (n=45)** | **No-Induction Group (n=103)** | **P-value†** |
| --- | --- | --- | --- |
| **Prescribed total dose, median (IQR), Gy** | 60.00 (56.00-60.20) | 60.00 (56.00-60.00) | 0.815 |
| Range, Gy | 40.00-69.96 | 45.00-66.00 | - |
| **Number of fractions, median (IQR)** | 30 (28-30) | 30 (28-30) | 0.692 |
| Range | 6-33 | 20-33 | - |
| **Fractional dose, median (IQR), Gy** | 2.00 (2.00-2.14) | 2.00 (2.00-2.13) | 0.578 |
| Range, Gy | 1.80-5.00 | 1.80-2.40 | - |
| **Overall treatment time, median (IQR), days** | 39.0 (33.0-42.0) | 40.0 (34.0-42.0) | 0.421 |
| Range, days | 6-50 | 20-50 | - |

**†**P-values were calculated using the Mann-Whitney U test for continuous variables.
